# Delayed Presentation of Pericardial Tamponade Resulting From Permanent Pacemaker Lead Perforation

**DOI:** 10.7759/cureus.85036

**Published:** 2025-05-29

**Authors:** Mackenzie Prendergast, Damian Liebhardt, Brit Long

**Affiliations:** 1 Emergency Medicine, Brooke Army Medical Center, San Antonio, USA

**Keywords:** pacemaker complication, pacemaker lead perforation, pericardial effusion, pericardial tamponade, ventricle perforation

## Abstract

Permanent pacemaker placement may be associated with several complications. This case report describes an 86-year-old male with a history of pacemaker placement five weeks prior, who presented to the emergency department via ambulance with concerns for acute occlusive myocardial infarction on electrocardiogram. He was found to have pericardial tamponade secondary to the puncture of the right ventricular wall by the pacer wire. This case report represents a rare, life-threatening complication of pacemaker placement. Recognition of this complication by emergency physicians is imperative and can rapidly change management. Treatment of pericardial tamponade can vary depending on patient stability; however, it ultimately requires drainage either surgically or with drain placement. Patients with pacemaker lead migration require revision of their pacemaker to prevent future complications.

## Introduction

Emergency physicians frequently treat patients with pacemakers and must be adept at identifying both common and rare complications associated with these devices, such as the uncommon occurrence of pericardial tamponade. Pericardial tamponade is a life-threatening condition caused by the accumulation of fluid in the pericardial sac [1]. As intrapericardial pressures increase, there is subsequent compression of the right atrium and right ventricle, leading to impaired venous return and reduced cardiac output [1]. Hypotension, tachycardia, and shock will occur without early recognition and prompt intervention to relieve the increased pressure [1]. We discuss an uncommon presentation of pericardial tamponade secondary to right ventricular perforation five weeks after pacemaker placement in an 86-year-old male brought in by emergency medical services (EMS) originally with concern for acute occlusive myocardial infarction (OMI).

## Case presentation

**Figure 1:**
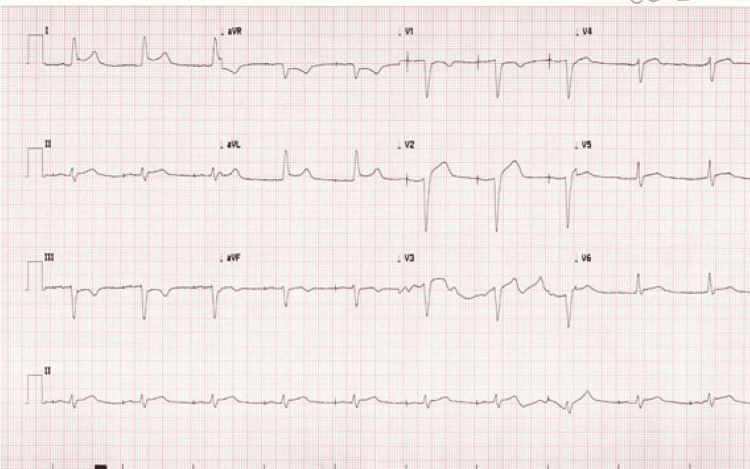
Initial electrocardiogram obtained on patient arrival in the emergency department demonstrating ST segment changes concerning for acute occlusive myocardial infarction.

An 86-year-old male with a history of atrial fibrillation on apixaban, hypertension, and new permanent pacemaker placement five weeks prior was transported by EMS as a “heart alert.” The patient had been traveling cross-country in his RV when he experienced a near-syncopal episode and chest pain. The EMS electrocardiogram (ECG) was concerning for occlusive myocardial infarction, and he was administered 324 mg chewable aspirin prior to arrival in the ED. Initial vital signs on arrival were notable for a blood pressure of 111/61 mmHg and a heart rate of 60 bpm, with telemetry demonstrating an atrioventricular paced rhythm. He appeared pale and diaphoretic, with a permanent pacemaker in the left chest wall and a well-healing surgical incision. ECG demonstrated left bundle branch block with ST elevations in leads I and aVL, and depressions in aVR, meeting Sgarbossa criteria (Figure 1).

**Figure 2:**
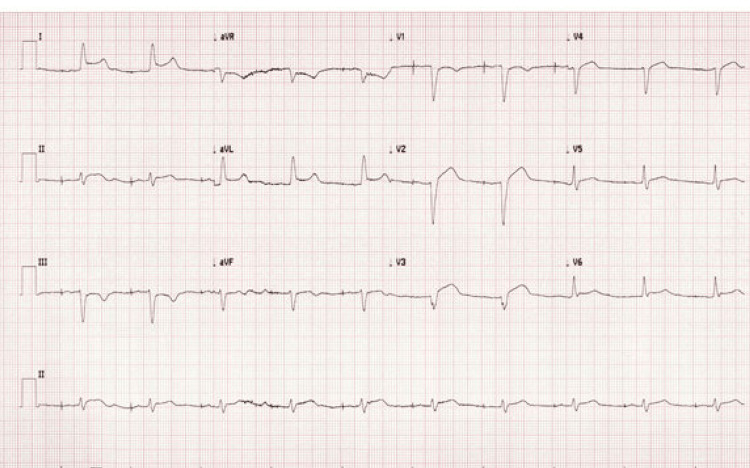
Repeat electrocardiogram obtained 15 minutes after initial ECG (see Figure 1) demonstrating more diffuse ST segment elevations.

The emergency physician emergently consulted the cardiologist and activated the catheterization lab. The patient then became hypotensive, with a blood pressure of 86/55 mmHg, and the emergency physician performed a point-of-care echocardiogram. This demonstrated large circumferential pericardial effusion with small right ventricle (RV) and right atrium (RA) with the presence of diastolic chamber collapse, consistent with tamponade physiology. He was administered a 250cc bolus of crystalloid, and the emergency physician also utilized push-dose phenylephrine. On repeat ECG, diffuse ST elevations were identified, but they were not consistent with any specific pattern of occlusion (Figure 2). This was thought to represent developing pericarditis or decreased global perfusion as a result of effusion with tamponade.

**Figure 3:**
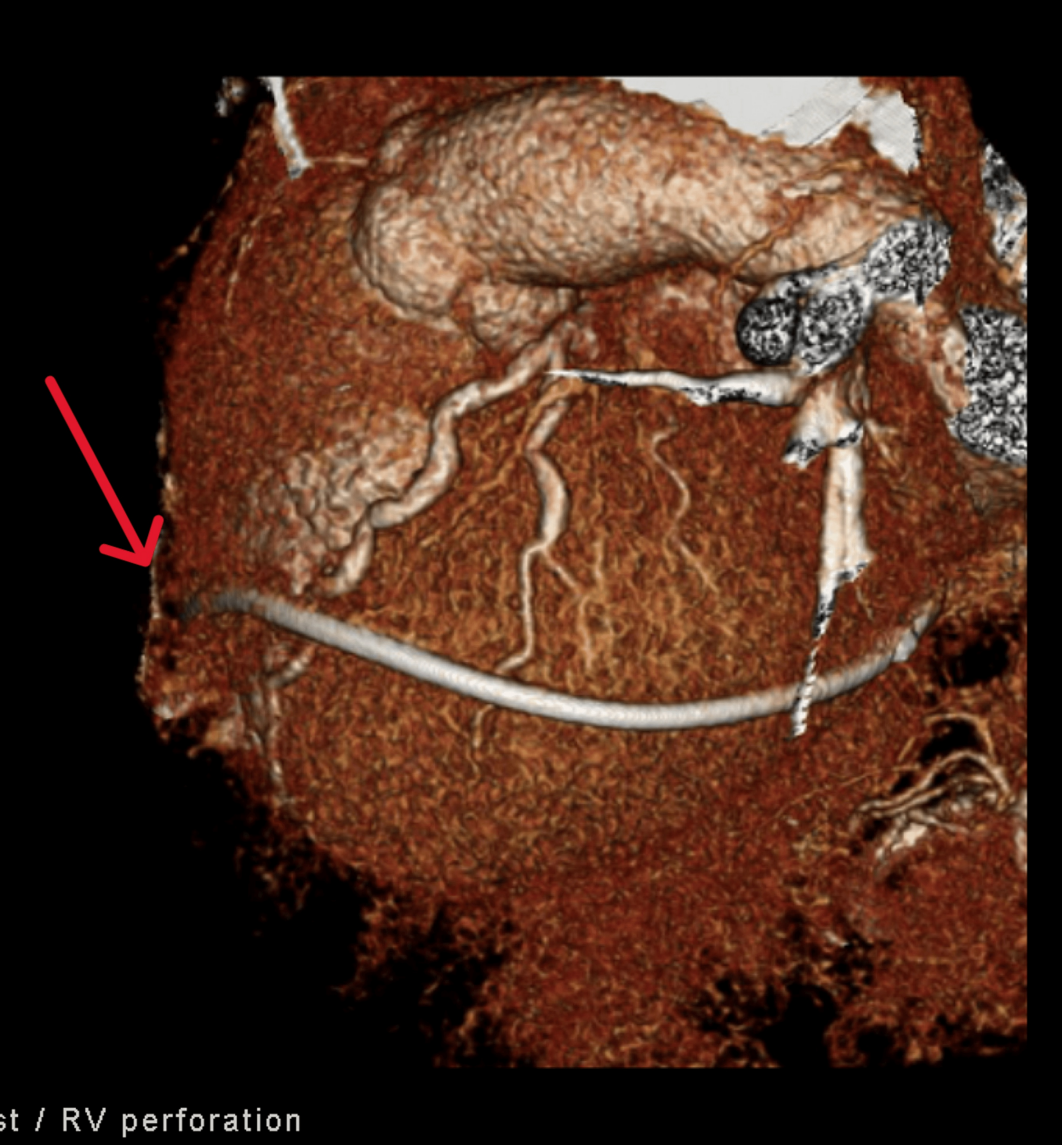
Three-dimensional reconstructed computed tomography image depicting right ventricular (RV) perforation with ventricular pacemaker lead wire (arrow).

**Figure 4:**
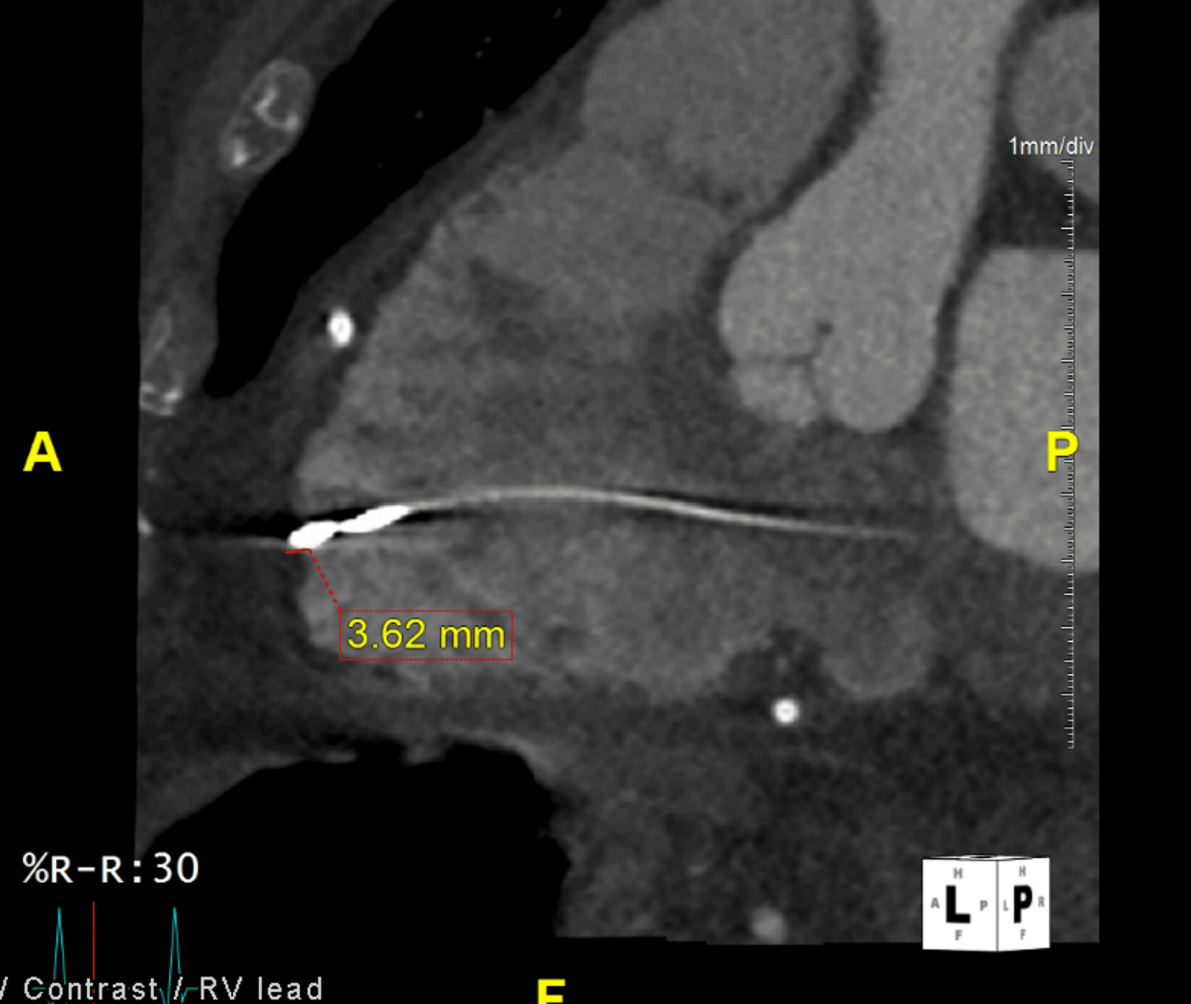
Computed tomography scan depicting right ventricular (RV) wire extending 3.62 mm through the wall of the RV.

The patient was taken emergently to the catheterization lab for pericardiocentesis, pericardial drain, and left heart catheterization. There was a total of 360cc sanguineous output after pericardiocentesis, and drain placement was completed by the cardiologist. His left heart catheterization did not demonstrate any coronary occlusion. Coronary computed tomography angiogram (CCTA) on hospital day two confirmed suspicion of RV perforation leading to tamponade with the RV lead tip perforating the RV, protruding approximately 3.5 mm beyond the RV free wall at the apex (Figures 3, 4). The patient ultimately underwent RV/RA pacemaker lead revision on hospital day five, followed by discharge on day 12.

## Discussion

Complications related to pacemakers may occur at any time during or after placement and at any point within the pacemaker system [2]. These complications may include the following: failure to pace, failure to capture, failure to sense, lead displacement, lead migration or fracture, infection, thrombophlebitis, pacemaker syndrome, and others [1,2]. In the immediate hours to days after placement, patients are at highest risk of pneumothorax, lead malpositioning, and ventricular perforation [3]. Literature suggests that pericardial effusion and tamponade are rare complications of pacemaker placement, occurring at an incidence of 0.8% and 0.5%, respectively [4]. Acute perforation typically presents within the first 24 hours of lead placement and is often identified during the procedure itself [3]. The majority of cases discussed in the literature regarding pacemaker lead perforation occur in the acute (within 24 hours) or subacute (1-30 days) period [5,6]. In contrast, delayed perforation, defined as occurring greater than one month post-procedure, is extremely uncommon [3,7]. One article discusses five cases of delayed lead perforation with subacute presentation occurring as late as 6-10 months post-procedure [6].

Rapid identification of complications is essential to patient care and may change emergency department management, as was the case in the scenario discussed above. Although this patient presented with concerns for decompensated OMI, he was determined to have pericardial tamponade with a need for emergent pericardial drainage. Standard treatments prior to percutaneous coronary intervention (i.e., heparin or other antithrombotic agent, sublingual nitroglycerin, etc.) would be contraindicated in such patients as they may worsen hemodynamics or bleeding. The use of diagnostic adjuncts, such as point-of-care ultrasound, can be instrumental in rapidly making this diagnosis. Patients with evidence of tamponade require emergent resuscitation and stabilization, along with pericardial drainage.

## Conclusions

This case highlights a rare but potentially life-threatening complication of pericardial tamponade secondary to RV perforation following pacemaker placement. While pacemaker-related complications are common, delayed perforation and tamponade occurring weeks after implantation are extremely unusual. Early recognition and prompt intervention are critical for improving patient outcomes. The present case emphasizes the timely use of diagnostic tools, such as point-of-care ultrasound, and early activation of the catheterization lab to make a rapid diagnosis and initiate timely management of tamponade, including pericardiocentesis and subsequent lead revision. Emergency physicians must maintain a high index of suspicion for such complications, particularly in patients with a recent pacemaker placement, as the clinical presentation can mimic more common conditions, such as myocardial infarction, and require pivoting from standard treatments. This case reinforces the importance of considering a broad differential diagnosis, rapid imaging, and tailored management strategies in patients presenting with pacemaker-related complications.
